# Concurrent adjacent strabismus surgery with glaucoma drainage device placement in childhood glaucomas

**DOI:** 10.1186/s12886-023-03275-8

**Published:** 2024-01-03

**Authors:** Adam Jacobson, Brenda L. Bohnsack

**Affiliations:** 1 Department of Ophthalmology and Visual Sciences, University of Michigan, 1000 Wall Street, Ann Arbor, MI 48105 USA; 2 Division of Ophthalmology, Ann & Robert H. Lurie Children’s Hospital of Chicago, 225 E. Chicago Ave, Chicago, IL 60611 USA; 3 Department of Ophthalmology, Northwestern University Feinberg School of Medicine, 645 N. Michigan Ave, Chicago, IL 60611 USA

**Keywords:** Strabismus, Exotropia, Esotropia, Childhood glaucomas, glaucoma drainage device

## Abstract

**Background:**

Determine outcomes of concurrent strabismus surgery with placement of a glaucoma drainage device (GDD) in children.

**Methods:**

Retrospective review of pediatric patients who underwent simultaneous lateral rectus (LR) muscle surgery with superotemporal GDD placement. Strabismus and GDD success were defined as residual horizontal misalignment < 10 prism diopter (PD) and intraocular pressure (IOP) < 21 mmHg, no visually devastating complications, and no additional IOP-lowering surgeries.

**Results:**

Fifteen eyes of 13 patients (69% male) underwent LR surgery (14 recessions, 1 resection) for exotropia or esotropia simultaneous with GDD placement (13 Baerveldt, 2 Ahmed) at 8.34 ± 5.26 years. Preoperative visual acuity (VA) in operative eye (0.89 ± 0.54) was worse than non-operative eye (0.23 ± 0.44, *p* = 0.0032). Preoperative horizontal deviation was 38.3 ± 9.4 PD and LR recession was 7.4 ± 1.1 mm. At final follow-up, VA in operative eye (0.87 ± 0.52) was unchanged from preoperative (*p* = 0.4062). Final IOP was significantly decreased (12.4 ± 4.7 mmHg vs. 31.1 ± 11.4 mmHg, *p* = 0.0001) as was number of glaucoma medications (2.7 ± 1.7 vs. 1.1 ± 1.3, *p* = 0.0037). Five (38%) and 9 patients (69%) met criteria for strabismus and GDD success, respectively. Two eyes required tube revision and endoscopic cyclophotocoagulation and 2 eyes had additional strabismus surgery.

**Conclusions:**

Concurrent strabismus and GDD surgery decreased horizontal deviation and obtained IOP control. It is important to consider correction of strabismus at time of GDD placement to maximize visual development and improve cosmesis in children with glaucoma.

## Background

Strabismus is common in children with glaucoma, especially in unilateral and asymmetric cases and early-onset eso- and exo- deviations can exacerbate vision loss from strabismic amblyopia [1–3]. While obtaining and maintaining intraocular pressure (IOP) control should be the primary focus, it is important to remember to treat amblyopia and strabismus in pediatric patients which may involve part-time occlusion, correction of refractive error, and strabismus surgery. Further, large angle deviations can be cosmetically and socially problematic particularly in teenagers and young adults, and strabismus has been well-established to be associated with mental health issues [4, 5].

Treatment of the strabismus in this setting is primarily surgical, however, glaucoma drainage devices (GDDs) can induce an additional restrictive component in both children and adults [1, 3, 6–9]. Additionally, placement of a GDD makes adjacent rectus muscle surgery more complicated and less predictable, often requiring hang-back sutures and removal of the capsule surrounding the plate [2, 7]. Nevertheless, concurrent strabismus surgery with GDD placement is not commonplace, which may be due to the need for expertise in both strabismus and glaucoma surgeries. Thus, there is limited information as to the success of both IOP control and eye alignment when same quadrant GDD and strabismus surgeries are combined.

## Methods

A retrospective case series identified pediatric patients (< 18 years old) who underwent concurrent strabismus surgery and GDD placement by one surgeon (BLB) at the Ann & Robert H. Lurie Children’s Hospital or the University of Michigan between 2011 and 2023. This study was approved as exempt without need for informed consent due to its retrospective nature by the Institutional Review Boards of the Ann & Robert H. Lurie Children’s Hospital and the University of Michigan. The study adhered to the tenets of the Declaration of Helsinki. Data collection was de-identified and HIPAA compliant.

Data collected included gender, race, ethnicity, age at time of surgery, ophthalmic and systemic diagnoses, and details of all strabismus and glaucoma surgeries. Childhood glaucomas were classified based on the World Glaucoma Association consensus [10]. Information obtained from the pre-operative and final examinations included best corrected visual acuity (BCVA), IOP, number of glaucoma medications, horizontal strabismus type in prism diopters (PD) in primary gaze at 20 feet, and ability to fuse or demonstrate stereopsis. Strabismus measurements were obtained by alternate prism cover testing if both eyes had BCVA such that the patient was able to fixate and refixate on at least a 20/400 optotype target. In cases where BCVA was worse than 20/400, strabismus measurements were obtained by Krimsky. All children were cooperative for optotype visual acuity (VA) testing (Allen, LEA, or Snellen), but fix and follow, count fingers, hand motions, and light perception were noted if BCVA was worse than the largest optotype. IOPs were measured by iCare (Revenue, Vantaa, Finland), Tonopen (Reichert, Depew, NY), or Goldmann application. Fusion was assessed by Worth 4 Dot and stereopsis was tested with standard Titmus stereotest with the fly, three animals, or nine circles.

**Fig. 1:**
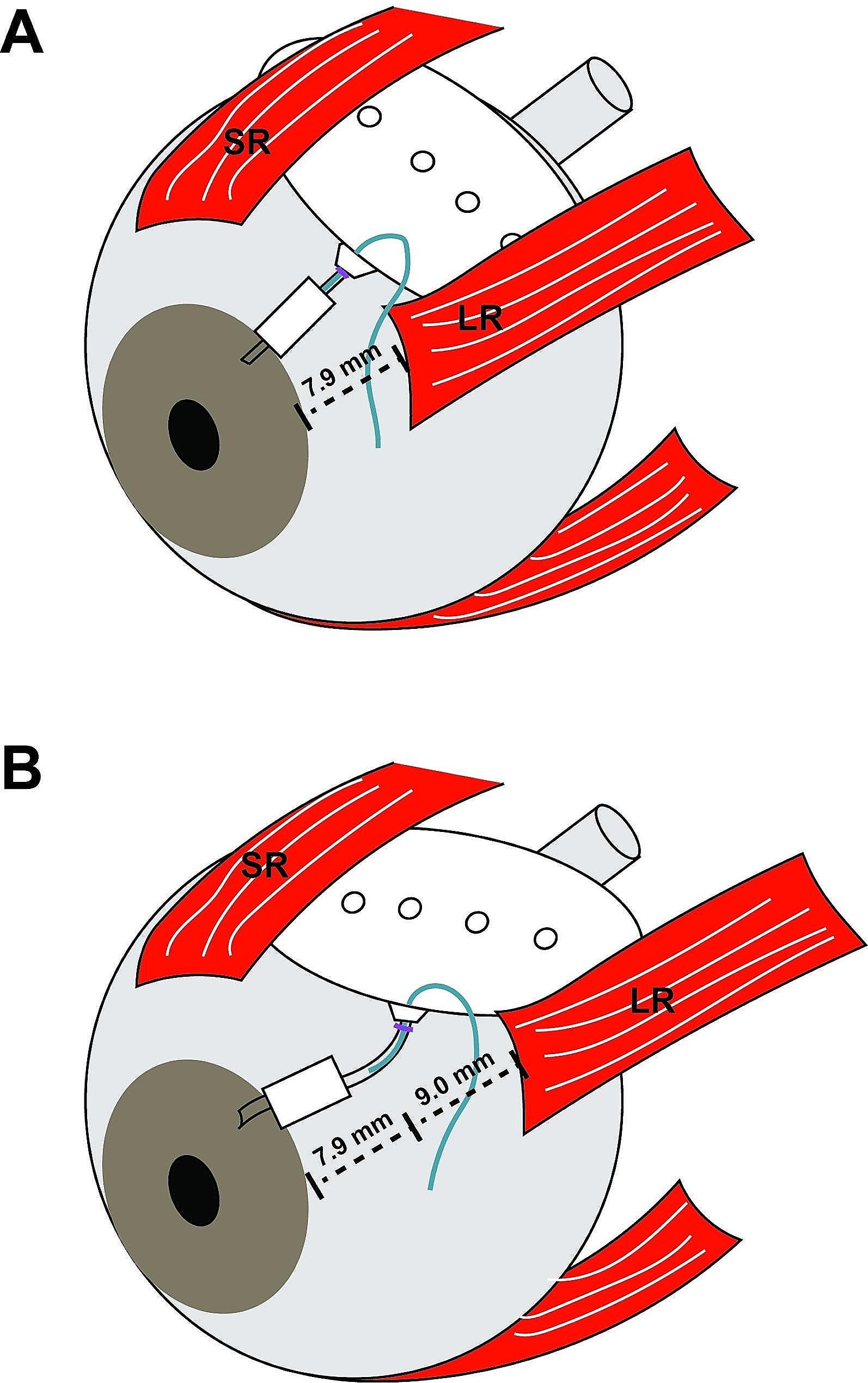
Schematic of Baerveldt 350 placement. (**A**) Typical placement of a Baerveldt 350 GDD approximately 8 mm posterior to the limbus such that the plate sits behind the insertions of the superior (SR) and an un-operated lateral rectus (LR) muscle. The plate lies along the equator of the globe, parallel to the limbus. (**B**) Placement of the plate of a Baerveldt 350 GDD behind a 9 mm recessed LR. In order to fit the plate behind the recessed muscle, the plate may need to be rotated such that it is not parallel to the limbus

Lateral rectus (LR) surgeries were performed from an inferotemporal fornix incision in order to not further disrupt the tenons capsule and conjunctiva in the superotemporal quadrant. The recession or resection amount was based on standard surgical dose charts [11]. In some eyes that underwent Baerveldt GDD placement, the amount of LR recession was decreased from the standard surgical dose chart in order to insure that the implant would have enough space posterior to the recessed insertion. No adjustments were made due to buphthalmos. The technique for Baerveldt and Ahmed implants was previously described using a superotemporal limbal-based incision [12, 13]. Care was taken when hooking the LR muscle which had just been reattached to the globe to ensure that the muscle was not disrupted or detached. The plate of the GDD, especially with Baerveldt implants, was placed beneath the superior rectus and the recessed (Fig. 1) or resected LR. In four eyes, the Baerveldt was implanted as a staged procedure (Table 1) such that after LR recession and plate placement, the tube was placed underneath the plate prior to closure of the Tenons and conjunctiva. In the other 11 eyes, the tube was placed within the anterior chamber or pars plana and covered with a scleral patch graft. The choice of GDD was based on surgeon preference, globe size, preoperative and target IOP, and history of prior glaucoma surgeries.

**Table 1:** Diagnoses and prior surgeries

Pt	Age (yrs)	Glaucoma Diagnosis	Systemic Diagnosis	Prior Glaucoma Surgeries	Prior Strabismus Surgeries
**1 OD**	**17.7**	Non-Acquired Systemic (Sturge Weber)	Sturge Weber, Developmental Delay, Seizures	Trabeculotomy x 2, Cycloablation x 4	
**2 OS**	**3.2**	Primary (PCG)		Goniotomy	
**3 OD**	**4.6**	GFCS		Trabeculotomy	
**4 OS**	**14.7**	Acquired (Traumatic)			
**5 OS**	**2.9**	Primary (PCG)		Trabeculotomy x 2	
**6 OD**	**14.0**	GFCS	Developmental Delay		MR Recession and LR Resection
**7 OS**	**10.7**	Non-Acquired Ocular (Anterior Segment Dysgenesis)	Developmental Delay		
**8 OD** **8 OS**	**4.9** **4.9**	Primary (JOAG) Primary (JOAG)		Trabeculotomy Trabeculotomy	
**9 OD** **9 OS**	**4.1** **4.1**	Primary (PCG) Primary (PCG)			
**10 OS**	**7.8**	Acquired (Traumatic)			
**11 OS**	**14.6**	Acquired (Traumatic)			
**12 OD**	**3.9**	Non-Acquired Ocular (Aniridia)			
**13 OS**	**5.4**	Non-Acquired Ocular (Peters Anomaly)	Developmental Delay		MR Recession and LR Resection

Abbreviations: OD, right eye; OS, left eye; PCG, Primary congenital glaucoma; GFCS, Glaucoma following cataract surgery; JOAG, Juvenile open angle glaucoma; MR, Medial rectus; LR, Lateral rectus

Strabismus surgery success was defined as < 10 PD residual horizontal deviation. For patients with bilateral surgery, only 1 eye was included in the pre- and post- strabismus analysis. Success of GDD was IOP < 21 mmHg, no visually devastating complications, and no additional IOP-lowering surgeries.

Statistical analyses included Mann-Whitney and Wilcoxon Rank Sum test. Tests were performed with GraphPad Prism 10 (GraphPad, La Jolla, CA). All tests were 2-sided with *p*-values less than 0.05 considered statistically significant.

## Results

Fifteen eyes of 13 patients (9 males and 4 females) underwent concurrent LR surgery with GDD placement at 8.3 ± 5.3 years of age (median 5.4, range 2.9–17.7). The types of glaucoma and prior surgeries are detailed in Table 1.

Preoperatively, 7 eyes had BCVA worse than the largest optotype and were noted as fix and follow, count fingers, hand motion or light perception. For the remaining 8 eyes, LogMAR BCVA (0.89 ± 0.54, median 1.23, range 0.18–1.30) was significantly worse than the non-operative eye (0.23 ± 0.44, median 0, range 0-1.3, *p* = 0.0032). Preoperative IOP (Table 2) was 31.1 ± 11.4 mmHg (median 28, range 12–47) and the number of glaucoma medications was 2.7 ± 1.7 (median 3, range 0–5). Although patient 12 had an IOP of 12 mmHg preoperatively, there were medication non-compliance issues, and he showed glaucoma progression with increased optic nerve cupping and myopic shift. Eleven patients showed a constant sensory exotropia (XT) while 1 patient had a basic pattern intermittent exotropia (X(T), Table 3). One patient had a sensory esotropia (ET). The average horizontal strabismus deviation was 38.5 ± 10.1 PD (median 40, range 20–50). Patient 8 who had X(T) was the only one to show preoperative fusion and stereopsis.

**Table 2:** Glaucoma surgery details

Pt	Preop IOP (mmHg)	Preop Meds	Glaucoma Surgery	Postop IOP (mmHg)	Postop Meds	Additional Glaucoma Surgeries
**1 OD**	45	3	Ahmed FP8	19	2	None
**2 OS**	22	2	BV 350	8	0	None
**3 OD**	32	3	BV350 Stage 1, Trabeculotomy	15	2	None
**4 OS**	24	4	BV350, ECP	13	0	None
**5 OS**	24	3	Ahmed FP7 with Ologen	16	2	None
**6 OD**	24	4	BV350, PPV	9	2	None
**7 OS**	40	3	BV350	24	2	Tube Revision Cycloablation
**8 OD** ^**1**^ **8 OS** ^**1**^	21 19	5 5	BV350 BV350 Stage 1	11 10	2 2	None BV350 Stage 2
**9 OD** **9 OS**	45 47	0 0	BV350 Stage 1, Goniotomy BV350 Stage 1, Goniotomy	9 10	0 0	BV350 Stage 2 BV350 Stage 2
**10 OS**	28	4	BV350	6	0	None
**11 OS**	40	0	BV350	15	2	None
**12 OD**	12	2	BV350	12	0	None
**13 OS**	44	3	BV350, PPV	9	0	Tube Revision Cycloablation

**Table 3:** Strabismus surgery details

Pt	Preop Strab	Preop Deviation (PD)	Strabismus Surgery	Surgery Amount (mm)	Postop Strab	Postop Deviation (PD)	Additional Strabismus Surgeries
**1 OD**	Sensory XT	40	LR Recession	10	XT	18	None
**2 OS**	Sensory XT	30	LR Recession	7	X(T)	8	None
**3 OD**	Sensory XT	50	LR Recession + MR Resection	8	ET	25	None
**4 OS**	Sensory XT	30	LR Recession + MR Resection	7	ET	10	None
**5 OS**	Sensory ET	30	LR Resection	8	ET	8	MR Recession
**6 OD**	Sensory XT	20	LR Recession	5	Ortho	0	MR Re-Recession
**7 OS**	Sensory XT	45	LR Recession	8	XT	35	None
**8 OD** ^**1**^ **8 OS** ^**1**^	Basic X(T) Basic X(T)	35 35	LR Recession LR Recession	7.5 7.5	X X	6 6	None None
**9 OD** **9 OS**	SensoryXT SensoryXT	40 40	LR Recession LR Recession	7 7	ET ET	25 25	None None
**10 OS**	Sensory XT	30	LR Recession	7	NA	NA	None
**11 OS**	Sensory XT	50	LR Recession	8	XT	15	None
**12 OD**	Sensory XT	50	LR Recession	7	ET	10	None
**13 OS**	Sensory XT	50	LR Recession	8	XT	1	None

Fourteen eyes of 12 patients underwent LR recession of which 2 patients also had ipsilateral medial rectus resection (Table 3). Two eyes of 2 patients who had a history of prior LR resection underwent recession of the previously resected muscle. The average recession was 7.4 ± 1.1 mm (median 7.3, range 5–10). Thirteen of these eyes had placement of a Baerveldt 350 GDD (Tables 2 and 9 as complete implantation, 4 as stage 1 placement). Six eyes had additional concurrent intraocular surgery including goniotomy (2), trabeculotomy, endoscopic cyclophotocoagulation, and pars plana vitrectomy for posterior placement of the tube (2). Patient 9 had late diagnosis PCG and underwent bilateral concurrent goniotomy with stage 1 Baerveldt placement due to the high risk of angle surgery failure. In the 2 patients who had bilateral surgery, LR recession and Baerveldt 350 placement was performed on both eyes on the same day. One eye had an Ahmed FP8 placed due to a history of extensive cycloablation. The one eye which was ET preoperatively, underwent an 8 mm LR resection and placement of Ahmed FP7 with Ologen augmentation. None of the eyes showed evidence of a slipped or lost muscle.

At final examination (2.6 ± 2.1 years, median 2.8, 0.04–5.6), 4 eyes had BCVA of count fingers, hand motion or light perception. The LogMAR BCVA of the remaining eyes (0.87 ± 0.52, median 1, range 0-1.6) was no different than preoperatively (*p* = 0.4062) and continued to be worse than the non-operative eye (0.24 ± 0.51, median 0, range 0-1.602, *p* = 0.0042) at final follow-up. IOP at final follow-up was significantly decreased (Tables 1 and 22.4 ± 4.7 mmHg, median 12, range 8–24, *p* = 0.0001) as was the number of glaucoma medications (1.1 ± 1.3, median 2, range 0–3, *p* = 0.0037) compared to preoperatively. Six patients had an exodeviation of which 4 eyes had constant deviation and 2 were intermittent or phoric (Table 3). Five patients had a constant ET. One patient was orthophoric and the deviation for 1 patient was not documented.

Five patients (38%), one of which had combined LR recession and MR resection, met the definition of strabismus success with the concurrent strabismus and GDD surgery (Table 3). One patient who underwent recession of a previously resected LR, reverted to esodeviation and underwent re-recession of the MR. The one patient who underwent LR resection had subsequent MR recession for continued ET. Ten eyes (67%) of 9 patients (69%) showed GDD success with no additional glaucoma surgeries or visually devastating complications (Table 2). Three eyes of 2 patients underwent stage 2 placement of the Baerveldt implant. If the stage 2 placement was considered an extension of the stage 1 surgery, then the success rate was increased to 87%. Two eyes of 2 patients required tube revision with endoscopic cyclophotocoagulation to obtain IOP control.

## Discussion

Strabismus is common in children with glaucoma affecting between 14 and 47% and can exacerbate amblyopia leading to worse visual outcomes [1, 3]. Amblyopia can be as much of a factor in visual prognosis in young children with glaucoma as controlling the IOP [14]. In addition, in later childhood and adolescence, eye misalignment often causes cosmetic and social concerns, and strabismus regardless of the etiology has been associated with mental health issues and worse self-esteem [4, 5].

Lateral rectus muscle surgery was performed at the time of superotemporal GDD placement if there was a moderate to large angle exotropia or esotropia. Concurrent surgery was performed to mainly decrease the need for operating on the lateral rectus muscle after GDD placement, which would be more complicated, less predictable, and could compromise the IOP-lowering effect of the GDD. While surgery could be done on the non-adjacent medial rectus instead of the lateral rectus, it is less likely that surgery only on the medial rectus muscle would yield enough improvement, especially with exotropias, where a medial rectus resection is more powerful when paired with a weakened (recessed) lateral rectus muscle. In addition, medial rectus surgery could be performed after the concurrent lateral rectus and GDD surgery, especially in cases of large-angle strabismus.

Despite the frequency of strabismus in children with glaucoma, to the best of our knowledge there has only been 1 other report of simultaneous strabismus surgery with GDD placement [2]. In Lee et al., 14 children had combined strabismus-GDD surgery and similar to our study the majority had LR recession for XT. However, their study had increased heterogeneity of selected GDD (Baerveldt 350, Baerveldt 250 and Ahmed FP7) and implant location (superotemporal, inferotemporal and inferonasal). Nevertheless, as in our study, strabismus surgery involved an adjacent rectus muscle, with similar amounts of recession. Both studies showed modest success rates for strabismus surgery (29% vs. 38% patients) while 11 of the 14 patients in Lee et al. had a postoperative decrease in horizontal deviation, all of the patients in our study had decreased angle postoperatively [2]. It is not surprising that the improvement in alignment did not coincide with better VA in our study. This is likely due to the contribution of other factors such as dense amblyopia and severe optic neuropathy.

It is important to note that in our study the addition of strabismus surgery did not hamper obtaining IOP control in our study. The IOP and number of glaucoma medications were significantly decreased at final follow-up compared to preoperatively. In contrast, Lee et al. did not include GDD success or IOP effect.2 In our study, 10 eyes (67%) did not require additional glaucoma surgery. Of the eyes that underwent additional glaucoma surgery, 3 of these had stage 2 placement of a Baerveldt whose plate was implanted at the time of LR recession. In the 4 eyes that had Stage 1 placement at the time of LR recession, 3 had concurrent angle surgery (trabeculotomy (1), goniotomy (2)). The reason for a staged procedure was that based on age and type of glaucoma, these eyes were deemed at high risk for angle surgery failure and placement of the Baerveldt plate would allow for full tube functionality upon stage 2 placement. One eye had a stage 1 Baerveldt placed at the same time as the other eye underwent a non-staged Baerveldt implant. This was to honor the parent’s wish to avoid same day bilateral intraocular surgery. In 3 of these eyes, the stage 2 placement was performed 4–5 weeks after the initial surgery, while the fourth eye has not required placement of the intraocular portion. Two additional eyes required tube revision (extension due to tube retraction (patient 7) and removal of vitreous occlusion despite concurrent PPV at time of GDD placement (patient 13)) and endoscopic cyclophotocoagulation to obtain IOP control. If the stage 2 placement was treated as an extension of the stage 1 surgery, the success rate at final follow-up was then 87%. Although there is a wide range of follow-up time in this study, this success rate is consistent with other studies of Baerveldt and Ahmed implants in children [12, 15–19].

The lack of reports on concurrent strabismus surgery and GDD placement is most likely due to the parceling of these surgeries between pediatric ophthalmologists and glaucoma specialists. For the glaucoma specialist, while IOP control is the top priority, it is important to consider the strabismus during preoperative evaluation. Addressing the strabismus, especially an exodeviation, after a GDD is placed, is more complicated and less predictable due to scarring between the bleb, plate and muscle.2, 7 In addition, in order to fully release the overlying muscle, the capsule of the plate may need to be excised [2, 7]. This could lead to early post-operative hypotony, but result in eventual encapsulation with increased IOP due to stimulation of the wound healing response.

The limitations of this study include its retrospective nature and lack of a control group. The majority of the patients (9 of 13) underwent unilateral LR surgery at the time of GDD placement. A control group was not included as unilateral LR surgery in the absence of either ipsilateral MR (e.g. LR recession and MR resection) or contralateral LR surgery (e.g. bilateral LR recession) was rarely performed by the authors in other cases of strabismus. Further, the study included a small number of patients that had various types of glaucoma, a range of horizontal deviations, and variable length of follow-up. Nevertheless, this study showed that same quadrant LR surgery at the same time as GDD placement did not compromise IOP control.

## Data Availability

Datasets during the current study are not publicly available, but are available from the corresponding author on reasonable request.

## Abbreviations

BCVA: best corrected visual acuity
BV350: Baerveldt 350 glaucoma drainage device
CF: count fingers
ECP: endoscopic cyclophotocoagulation
ET: esotropia
F&F: Fix and follow
GDD: glaucoma drainage device
GFCS: glaucoma following cataract surgery
HM: hand motions
IOP: intraocular pressure
JOAG: juvenile open angle glaucoma
LP: light perception
LR: lateral rectus
MR: medial rectus
NA: not available
OD: right eye
OS: left eye
Ortho: Orthophoric
PCG: primary congenital glaucoma
PD: prism diopter
PPV: pars plana vitrectomy
XT: exotropia
X(T): intermittent exotropia
VA: visual acuity
